# Changes in amount and intensity of physical activity over time in breast cancer survivors

**DOI:** 10.1093/jncics/pkad056

**Published:** 2023-08-10

**Authors:** Paulina S Marell, Robert A Vierkant, Janet E Olson, Joerg Herrmann, Nicole L Larson, Nathan K Lebrasseur, Stacy D D’Andre, Diane K Ehlers, Daniela L Stan, Andrea L Cheville, Toure Barksdale, Charles L Loprinzi, Fergus J Couch, Kathryn J Ruddy

**Affiliations:** Community Internal Medicine, Geriatrics, and Palliative Care, Mayo Clinic, Rochester, MN, USA; Quantitative Health Sciences, Mayo Clinic, Rochester, MN, USA; Quantitative Health Sciences, Mayo Clinic, Rochester, MN, USA; Cardiovascular Medicine, Mayo Clinic, Rochester, MN, USA; Quantitative Health Sciences, Mayo Clinic, Rochester, MN, USA; Physical Medicine and Rehabilitation, Mayo Clinic, Rochester, MN, USA; Oncology, Mayo Clinic, Rochester, MN, USA; Quantitative Health Sciences, Mayo Clinic, Phoenix, AZ, USA; General Internal Medicine, Mayo Clinic, Rochester, MN, USA; Physical Medicine and Rehabilitation, Mayo Clinic, Rochester, MN, USA; Physical Medicine and Rehabilitation, Mayo Clinic, Rochester, MN, USA; Oncology, Mayo Clinic, Rochester, MN, USA; Laboratory Medicine and Pathology, Mayo Clinic, Rochester, MN, USA; Oncology, Mayo Clinic, Rochester, MN, USA

## Abstract

**Background:**

Physical activity is associated with decreased breast cancer recurrence and mortality, as well as fewer treatment-related symptoms. Nevertheless, most breast cancer survivors do not meet physical activity guidelines. The purpose of this manuscript is to characterize physical activity trends over time in breast cancer survivors.

**Methods:**

Mayo Clinic Breast Disease Registry participants received surveys at baseline and at 1 and 4 years after diagnosis; breast cancer recurrence and/or metastatic disease were exclusion criteria. Participants were considered to be meeting guidelines if they self-reported at least 150 minutes of moderate-intensity (eg, fast walking) and/or strenuous (eg, jogging) physical activity per week. Statistical analyses include analysis of covariance methods, paired *t* tests, conditional logistic regression models, and McNemar tests of homogeneity.

**Results:**

A total of 171 participants were included in the analysis. The amount of total physical activity decreased over time (*P* = .07). Mild-intensity physical activity (eg, easy walking) decreased most over time (*P* = .05). Among participants aged 18-49 years, mild-intensity (*P* = .05) and moderate-intensity (*P* = .02) physical activity decreased over time. Strenuous-intensity physical activity levels decreased over time among participants with a normal body mass index (*P* = .002) and with obesity (*P* = .01).

**Conclusions:**

We found a trend-level decrease in total physical activity over time, driven mostly by a decrease in mild-intensity physical activity. Young breast cancer survivors are especially likely to reduce their physical activity over time. Further research on implementing physical activity guidelines in clinical practice is warranted.

In 2023, more than 290 000 people are expected to be newly diagnosed with breast cancer in the United States, comprising 31% of new cancer diagnoses in women. The 5-year survival rate for invasive breast cancer is approximately 90% and rising, such that the population of breast cancer survivors is large (nearly 4 million in the United States) and growing (1).

Previous research has shown that higher physical activity levels decrease the risk of many cancers, including female breast cancer (2-4). Additionally, physical activity decreases the likelihood of experiencing adverse treatment effects, resulting in better bone and cognitive health, less sexual dysfunction, vasomotor symptoms, lymphedema, anxiety, fatigue, and better overall quality of life (5-7). Importantly, physical activity also has been associated with a decreased likelihood of breast cancer recurrence, cancer-related mortality, and all-cause mortality (5,8-13), and there are dose-response relationships between overall and cancer-specific mortality and exercise duration and intensity (14,15).

The American Cancer Society, National Comprehensive Cancer Network, American College of Sports Medicine, and US Department of Health and Human Services recommend that adults engage in at least 150 minutes of moderate-intensity and/or strenuous physical activity each week, in addition to muscle-strengthening exercises (1,3,7,16). According to data from the 2013-2017 National Health Interview Survey, 14.2% of cancer survivors vs 21.1% of adults without a history of cancer met these guidelines, with 39.2% of cancer survivors vs 31.2% of those without a history of cancer reporting no physical activity (17). Specifically, breast cancer survivors have been found to be more sedentary and participate less in mild-intensity physical activity relative to the general population (18), and a previous study identified that the percentage of those meeting guidelines declines over time after breast cancer treatment (19). Physical activity intensity decreases for many patients during cancer-directed therapies (20). Among women diagnosed with ductal carcinoma in situ, undergoing a unilateral or bilateral mastectomy and endorsing anxiety at enrollment were associated with decreased exercise levels at the 18-month follow-up time relative to baseline (21). Furthermore, breast cancer survivors who were classified as fatigued after completing treatment had higher levels of persistent cancer-related fatigue and lower levels of physical fitness at least 5 years after initial diagnosis (22). Based on survey data from breast cancer survivors, our group has previously shown that younger age, absence of recurrent breast cancer, not undergoing a bilateral mastectomy, absence of metastatic disease, and lower body mass index (BMI) at the time of survey completion were associated with meeting physical activity guidelines (23). As much evidence supports the positive effects of physical activity in breast cancer survivors, additional research to characterize physical activity longitudinally and determine factors that might put a survivor at elevated risk of declining physical activity over the years following a breast cancer diagnosis will help inform and target future interventions to increase physical activity. Thus, the current project sought to characterize trends in amount and intensity of physical activity over time in breast cancer survivors using data from the Mayo Clinic Breast Disease Registry.

## Methods

Adults seen at least once at Mayo Clinic Rochester for breast cancer who were diagnosed within the year prior were invited to prospectively enroll in the Mayo Clinic Breast Disease Registry. Those who signed informed consent were asked to complete a baseline survey and then annual follow-up surveys, some of which asked about physical activity. This current manuscript uses data from baseline surveys mailed between 2015 and 2016, year 1 surveys mailed between 2015 and 2020 (approximately 1 year after a breast cancer diagnosis), and year 4 surveys mailed between 2017 and 2020 (approximately 4 years after a breast cancer diagnosis). The surveys included a modified Godin Leisure-Time Exercise Questionnaire (24) in which participants were asked to report the minutes per day and days per week in which they engaged in mild-intensity, moderate-intensity, and/or strenuous physical activity. Mild-intensity physical activity was described as “minimal effort” and examples included yoga, bowling, and easy walking. Moderate-intensity physical activity was described as “not exhausted” with the following examples provided: tennis, easy swimming, and fast walking. Finally, strenuous physical activity was described as “heart beats rapidly,” and examples included jogging, vigorous biking, and cross-country skiing. Participants were asked to return their completed survey in a prestamped and pre-addressed envelope to the Mayo Clinic Breast Disease Registry study team. This prospective cohort study did not include any intervention, and no specific counseling was provided to patients as part of this study.

Participants were included in the analysis if they 1) completed a baseline survey between 2015 and 2016 (such that they had an opportunity to complete year 1 and year 4 surveys at the time of this analysis); 2) returned surveys with completed information regarding mild-intensity, moderate-intensity, and strenuous physical activity data on baseline, year 1, and year 4 surveys; and 3) had a baseline survey date that was collected at or less than 6 months after their diagnosis. Participants were excluded if they had documentation of breast cancer recurrence and/or metastatic disease at any time before the return of their year 4 survey. Participants were considered to be meeting guidelines if they self-reported at least 150 minutes of moderate-intensity and/or strenuous aerobic physical activity per week on average. Those who reported 150 minutes or more of only mild-intensity physical activity were not considered to be meeting guidelines.

We assessed the trends in average minutes per week of physical activity across the baseline, year 1, and year 4 surveys and the percentage of participants who were meeting guidelines across those same timepoints. We also examined the extent to which age at diagnosis and BMI were associated with physical activity levels at each timepoint and performed subgroup analyses of change in physical activity across timepoints by age and BMI categories. Associations of within-participant changes in minutes of physical activity per week across survey times were first examined using analysis of covariance (ANCOVA) methods. For these, we modeled minutes of physical activity per week as the outcome variable and survey time as a fixed exposure term, and we accounted for within-participant collinearity by modeling participant identifier as a fixed blocking term. Global tests using ANCOVA were followed by a series of pairwise comparisons using paired *t* tests, comparing changes in physical activity from baseline to year 1 survey, from baseline to year 4 survey, and from year 1 to year 4 survey. Separate analyses were carried out for mild-intensity, moderate-intensity, strenuous, and total minutes of physical activity. Associations of within-participant changes in meeting guidelines for 150 minutes or longer of moderate-intensity or strenuous physical activity per week across survey times were first examined using conditional logistic regression models. For these, we modeled meeting guidelines as the outcome variable and survey time as the exposure variable, and we accounted for within-participant collinearity by modeling participant identifier as a stratification (matched set) term. The logistic regression global test was followed by a series of pairwise comparisons of changes in meeting guidelines using McNemar tests of homogeneity. This longitudinal cohort study was approved by the Mayo Clinic institutional review board.

## Results

### Sample characteristics

**Figure 1.**
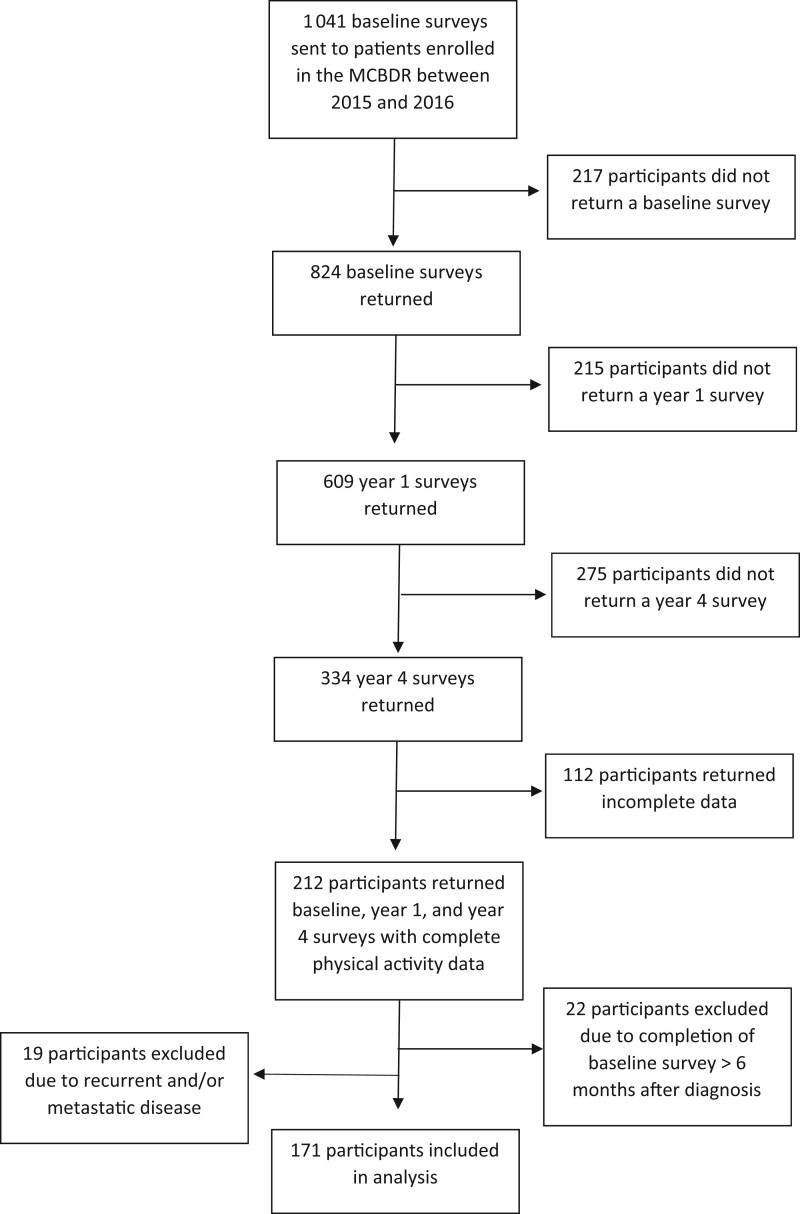
Participant flow diagram. MCBDR = Mayo Clinic Breast Disease Registry.

Of 1041 participants who were sent the baseline survey between 2015 and 2016, 824 returned the survey (79% response rate). Among these baseline survey respondents, 609 (74%) also returned a year 1 survey, and 334 (41%) returned both the year 1 and year 4 surveys. A total of 122 participants returned incomplete physical activity data, such that 212 participants met the inclusion guidelines. Twenty-two participants were excluded because of completion of the baseline survey more than 6 months after diagnosis, and an additional 19 participants were excluded because of documentation in the electronic medical records of recurrent breast cancer and/or metastatic disease at or before 4 years after diagnosis. A total of 171 participants were ultimately included in the analysis of physical activity trends (Figure 1). Of the 171 participants, 25% were aged 18-49 years when sent the baseline survey, 30% were aged 50-59 years, 26% were aged 60-69 years, and 19% were 70 years or older at baseline. Approximately 36% of participants were of a normal weight (BMI = 18.5-24.9 kg/m^2^), 35% were overweight (BMI = 25.0-29.9 kg/m^2^), and 29% were obese (BMI ≥ 30.0 kg/m^2^). Full details on the sample characteristics are provided in Table 1.

**Table 1.** Characteristics of survey respondents and compliance with physical activity guidelines

Characteristics	Baseline meeting guidelines for moderate or strenuous exercise				Year 1 meeting guidelines for moderate or strenuous exercise				Year 4 meeting guidelines for moderate or strenuous exercise				
	No (n = 104)	Yes (n = 67)	Total (n = 171)	*P* ^a^	No (n = 103)	Yes (n = 68)	Total (n = 171)	*P* ^a^		No (n = 109)	Yes (n = 62)	Total (n = 171)	*P* ^a^
Age at diagnosis, No. (%), y				<.001				.185					.102
18-39	5 (4.8)	12 (17.9)	17 (9.9)		13 (12.6)	4 (5.9)	17 (9.9)			12 (11.0)	5 (8.1)	17 (9.9)	
40-49	11 (10.6)	14 (20.9)	25 (14.6)		16 (15.5)	9 (13.2)	25 (14.6)			12 (11.0)	13 (21.0)	25 (14.6)	
50-59	26 (25.0)	25 (37.3)	51 (29.8)		26 (25.2)	25 (36.8)	51 (29.8)			28 (25.7)	23 (37.1)	51 (29.8)	
60-69	37 (35.6)	8 (11.9)	45 (26.3)		31 (30.1)	14 (20.6)	45 (26.3)			32 (29.4)	13 (21.0)	45 (26.3)	
70 and older	25 (24.0)	8 (11.9)	33 (19.3)		17 (16.5)	16 (23.5)	33 (19.3)			25 (22.9)	8 (12.9)	33 (19.3)	
Gender, No. (%)				.521				.518					.535
Female	102 (98.1%)	67 (100.0)	169 (98.8)		101 (98.1)	68 (100.0)	169 (98.8)			107 (98.2)	62 (100.0)	169 (98.8)	
Male	2 (1.9)	0 (0.0)	2 (1.2)		2 (1.9)	0 (0.0)	2 (1.2)			2 (1.8)	0 (0.0)	2 (1.2)	
Race, No. (%)				.458				.797					.147
African, African American, Black	2 (1.9)	0 (0.0)	2 (1.2)		2 (1.9)	0 (0.0)	2 (1.2)			2 (1.8)	0 (0.0)	2 (1.2)	
Other	2 (1.9)	2 (3.0)	4 (2.3)		2 (1.9)	2 (2.9)	4 (2.3)			4 (3.7)	0 (0.0)	4 (2.3)	
Unknown, choose not to provide	0 (0.0)	1 (1.5)	1 (0.6)		1 (1.0)	0 (0.0)	1 (0.6)			0 (0.0)	1 (1.6)	1 (0.6)	
White	100 (96.2)	64 (95.5)	164 (95.9)		98 (95.1)	66 (97.1)	164 (95.9)			103 (94.5)	61 (98.4)	164 (95.9)	
Ethnicity, No. (%)				.367				.559					.573
Hispanic, Latino	1 (1.0)	2 (3.0)	3 (1.8)		2 (1.9)	1 (1.5)	3 (1.8)			2 (1.8)	1 (1.6)	3 (1.8)	
Not Hispanic, Latino	102 (98.1)	63 (94.0)	165 (96.5)		98 (95.1)	67 (98.5)	165 (96.5)			106 (97.2)	59 (95.2)	165 (96.5)	
Unknown, choose not	1 (1.0)	2 (3.0)	3 (1.8)		3 (2.9)	0 (0.0)	3 (1.8)			1 (0.9)	2 (3.2)	3 (1.8)	
BMI, No. (%)				<.001				.903					<.001
18.5-24.9 kg/m^2^	27 (26.2)	34 (51.5)	61 (36.1)		36 (35.3)	25 (37.3)	61 (36.1)			27 (24.8)	34 (56.7)	61 (36.1)	
25.0-29.9 kg/m^2^	34 (33.0)	25 (37.9)	59 (34.9)		35 (34.3)	24 (35.8)	59 (34.9)			42 (38.5)	17 (28.3)	59 (34.9)	
≥30.0 kg/m^2^	42 (40.8)	7 (10.6)	49 (29.0)		31 (30.4)	18 (26.9)	49 (29.0)			40 (36.7)	9 (15.0)	49 (29.0)	
Missing	1	1	2		1	1	2			0	2	2	
Highest level of education, No. (%)				.009				.796					.828
Bachelor degree	22 (21.2)	27 (40.3)	49 (28.7)		30 (29.1)	19 (27.9)	49 (28.7)			29 (26.6)	20 (32.3)	49 (28.7)	
Graduate school	29 (27.9)	21 (31.3)	50 (29.2)		32 (31.1)	18 (26.5)	50 (29.2)			33 (30.3)	17 (27.4)	50 (29.2)	
High school graduate, GED	17 (16.3)	2 (3.0)	19 (11.1)		9 (8.7)	10 (14.7)	19 (11.1)			14 (12.8)	5 (8.1)	19 (11.1)	
Some college or associate degree but not bachelor degree	28 (26.9)	13 (19.4)	41 (24.0)		25 (24.3)	16 (23.5)	41 (24.0)			26 (23.9)	15 (24.2)	41 (24.0)	
Vocational education beyond high school	8 (7.7)	4 (6.0)	12 (7.0)		7 (6.8)	5 (7.4)	12 (7.0)			7 (6.4)	5 (8.1)	12 (7.0)	
Hypercholesterolemia, No. (%)				.068				.410					.095
No	62 (60.2)	50 (74.6)	112 (65.9)		70 (68.6)	42 (61.8)	112 (65.9)			66 (61.1)	46 (74.2)	112 (65.9)	
Yes	41 (39.8)	17 (25.4)	58 (34.1)		32 (31.4)	26 (38.2)	58 (34.1)			42 (38.9)	16 (25.8)	58 (34.1)	
Missing	1	0	1		1	0	1			1	0	1	
Heart disease, No. (%)				1.000				.156					.490
No	95 (94.1)	62 (95.4)	157 (94.6)		98 (97.0)	59 (90.8)	157 (94.6)			99 (93.4)	58 (96.7)	157 (94.6)	
Yes	6 (5.9)	3 (4.6)	9 (5.4)		3 (3.0)	6 (9.2)	9 (5.4)			7 (6.6)	2 (3.3)	9 (5.4)	
Missing	3	2	5		2	3	5			3	2	5	
Hypertension, No. (%)				.012				.615					.089
No	61 (59.2)	51 (78.5)	112 (66.7)		66 (64.7)	46 (69.7)	112 (66.7)			66 (61.7)	46 (75.4)	112 (66.7)	
Yes	42 (40.8)	14 (21.5)	56 (33.3)		36 (35.3)	20 (30.3)	56 (33.3)			41 (38.3)	15 (24.6)	56 (33.3)	
Missing	1	2	3		1	2	3			2	1	3	
Stroke, No. (%)				.283				.563					.555
No	99 (97.1)	65 (100.0)	164 (98.2)		100 (99.0)	64 (97.0)	164 (98.2)			105 (99.1)	59 (96.7)	164 (98.2)	
Yes	3 (2.9)	0 (0.0)	3 (1.8)		1 (1.0)	2 (3.0)	3 (1.8)			1 (0.9)	2 (3.3)	3 (1.8)	
Missing	2	2	4		2	2	4			3	1	4	
Diabetes, No. (%)				<.001				1.000					.012
No	84 (82.4)	64 (98.5)	148 (88.6)		89 (88.1)	59 (89.4)	148 (88.6)			89 (84.0)	59 (96.7)	148 (88.6)	
Yes	18 (17.6)	1 (1.5)	19 (11.4)		12 (11.9)	7 (10.6)	19 (11.4)			17 (16.0)	2 (3.3)	19 (11.4)	
Missing	2	2	4		2	2	4			3	1	4	
Lung disease, No. (%)				.120				.662					.073
No	82 (81.2)	59 (90.8)	141 (84.9)		86 (86.0)	55 (83.3)	141 (84.9)			85 (81.0)	56 (91.8)	141 (84.9)	
Yes	19 (18.8)	6 (9.2)	25 (15.1)		14 (14.0)	11 (16.7)	25 (15.1)			20 (19.0)	5 (8.2)	25 (15.1)	
Missing	3	2	5		3	2	5			4	1	5	
Liver disease, No. (%)				1.000				1.000					.555
No	100 (98.0)	64 (98.5)	164 (98.2)		99 (98.0)	65 (98.5)	164 (98.2)			105 (99.1)	59 (96.7)	164 (98.2)	
Yes	2 (2.0)	1 (1.5)	3 (1.8)		2 (2.0)	1 (1.5)	3 (1.8)			1 (0.9)	2 (3.3)	3 (1.8)	
Missing	2	2	4		2	2	4			3	1	4	
Kidney disease, No. (%)				.082				1.000					.416
No	95 (94.1)	65 (100.0)	160 (96.4)		96 (96.0)	64 (97.0)	160 (96.4)			100 (95.2)	60 (98.4)	160 (96.4)	
Yes	6 (5.9)	0 (0.0)	6 (3.6)		4 (4.0)	2 (3.0)	6 (3.6)			5 (4.8)	1 (1.6)	6 (3.6)	
Missing	3	2	5		3	2	5			4	1	5	
Surgery type, No. (%)				<.001				.816					.011
Bilateral mastectomy	24 (23.1)	34 (50.7)	58 (33.9)		37 (35.9)	21 (30.9)	58 (33.9)			29 (26.6)	29 (46.8)	58 (33.9)	
Biopsy only, other, unknown	10 (9.6)	3 (4.5)	13 (7.6)		7 (6.8)	6 (8.8)	13 (7.6)			7 (6.4)	6 (9.7)	13 (7.6)	
Lumpectomy	54 (51.9)	16 (23.9)	70 (40.9)		40 (38.8)	30 (44.1)	70 (40.9)			54 (49.5)	16 (25.8)	70 (40.9)	
Unilateral mastectomy	16 (15.4)	14 (20.9)	30 (17.5)		19 (18.4)	11 (16.2)	30 (17.5)			19 (17.4)	11 (17.7)	30 (17.5)	
Radiotherapy, No. (%)				.007				.754					.011
No	38 (36.5)	39 (58.2)	77 (45.0)		45 (43.7)	32 (47.1)	77 (45.0)			41 (37.6)	36 (58.1)	77 (45.0)	
Yes	66 (63.5)	28 (41.8)	94 (55.0)		58 (56.3)	36 (52.9)	94 (55.0)			68 (62.4)	26 (41.9)	94 (55.0)	
Chemotherapy, No. (%)				.731				.122					.600
No	75 (72.1)	46 (68.7)	121 (70.8)		68 (66.0)	53 (77.9)	121 (70.8)			79 (72.5)	42 (67.7)	121 (70.8)	
Yes	29 (27.9)	21 (31.3)	50 (29.2)		35 (34.0)	15 (22.1)	50 (29.2)			30 (27.5)	20 (32.3)	50 (29.2)	
Hormone therapy, No. (%)				.747				.748					.570
No	44 (42.3)	24 (35.8)	68 (39.8)		41 (39.8)	27 (39.7)	68 (39.8)			42 (38.5)	26 (41.9)	68 (39.8)	
Unknown	2 (1.9)	1 (1.5)	3 (1.8)		1 (1.0)	2 (2.9)	3 (1.8)			3 (2.8)	0 (0.0)	3 (1.8)	
Yes	58 (55.8)	42 (62.7)	100 (58.5)		61 (59.2)	39 (57.4)	100 (58.5)			64 (58.7)	36 (58.1)	100 (58.5)	

a Fisher exact *P* value. BMI = body mass index; GED = general educational development.

At the baseline timepoint, younger age at diagnosis, lower BMI group, higher level of education, undergoing a bilateral mastectomy, not receiving radiotherapy, and self-reported comorbidities of hypertension and diabetes had statistically significant associations with meeting physical activity guidelines (Table 1). At the year 1 timepoint, no participant characteristics had statistically significant associations (Table 1). At the year 4 timepoint, only lower BMI group, type of surgery undergone (bilateral mastectomy), not receiving radiotherapy, and self-reported comorbidity of diabetes on the baseline survey had statistically significant associations with meeting physical activity guidelines (Table 1).

There was no statistical difference over time in the percentage of participants meeting the 150 minutes or more guideline for moderate-intensity and/or strenuous activity: 39% at baseline, 40% at year 1, and 36% at year 4 (Table 2). Approximately one-third (n = 51, 29.8%) of participants never met physical activity guidelines at any timepoint. A minority of participants were consistent in their guideline adherence, with only 18 (10.5%) meeting the guideline across the 3 timepoints. Those who never met guidelines were more likely to have an elevated BMI (*P* = .001) relative to those who always met guidelines (Supplementary Table 1, available online).

**Table 2.** Associations of changes in meeting guidelines for 150 minutes or longer of moderate and/or strenuous physical activity per week across survey times

Survey	No. meeting guidelines (%)	OR (95% CI)^a^^,^^b^	Global *P*^a^^,^^c^	Pairwise *P*^d^
			.74	.91, .48, .49
Baseline	67 (39)	1.00 (Referent)		
Year 1	68 (40)	1.03 (0.64 to 1.65)		
Year 4	62 (36)	0.86 (0.54 to 1.39)		

a Conditional logistic regression analysis, model meeting guidelines as the outcome variable, survey time as exposure variable, and subject as a stratification (matched set) term. CI = confidence interval; OR = odds ratio.

b Estimated increase in odds of meeting guidelines from baseline to year 1 and year 4 follow-up surveys, respectively.

c Global test assessing if meeting guidelines differed across any of the 3 survey times.

d McNemar tests of homogeneity assessing in proportion meeting guidelines at baseline survey compared with year 1 survey, at baseline survey compared with year 4 survey and at year 1 survey compared with year 4 survey.

### Trends in physical activity across timepoints

**Figure 2.**
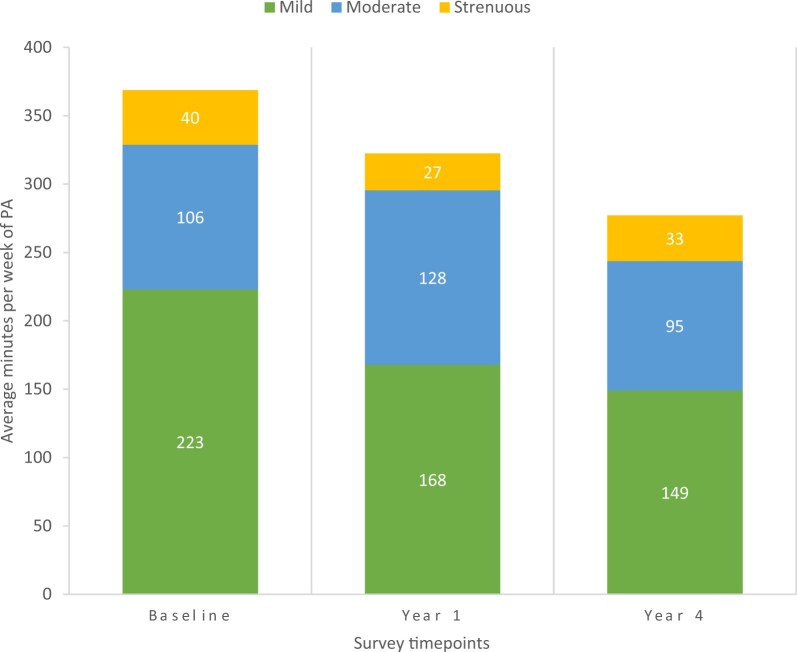
Average physical activity over time. Average minutes per week of mild-intensity, moderate-intensity, and strenuous physical activity at baseline, year 1, and year 4 timepoints. PA = physical activity.

When assessing the total amount of any type of physical activity, the average time spent in physical activity decreased over time, from 369 minutes/week at baseline to 322 minutes/week at year 1 follow-up to 277 minutes/week at year 4 follow-up, although these trends did not reach global statistical significance (ANCOVA *P* = .07; Table 3, Figure 2). Globally, only participation in mild-intensity physical activity was found to have a statistically significant decrease in average minutes per week over time (ANCOVA *P* = .05; Table 3). Using pairwise analyses, we found that the change across all physical activity intensity levels was not statistically significant when compared from baseline to year 1 and year 1 to year 4. However, comparing baseline to year 1, we found statistically significant decreases in mild-intensity (*P* = .03) and total (*P* = .01) physical activity levels (Table 3).

**Table 3.** Associations of changes in mild-intensity, moderate-intensity, strenuous, and total minutes of physical activity per week across survey times

Minutes of physical activity per week	Mean (SD)	Parameter estimate (SE)^a^^,^^b^	Global *P*^a^^,^^c^	Pairwise *P*^d^
Mild			.05	.14, .03, .36
Baseline	223 (459)	Referent		
Year 1	168 (207)	−55.1 (−115.8, 5.6)		
Year 4	149 (210)	−73.9 (−134.6, −13.2)		
Moderate			.30	.40, .39, .17
Baseline	106 (182)	Referent		
Year 1	128 (294)	21.7 (−20.4, 63.9)		
Year 4	95 (125)	−11.1 (−53.2, 31.1)		
Strenuous			.21	.10, .30, .42
Baseline	40 (89)	Ref		
Year 1	27 (69)	−13.4 (−28.2, 1.5)		
Year 4	33 (86)	−6.7 (−21.5, 8.2)		
Total			.07	.34, .01, .16
Baseline	369 (573)	Referent		
Year 1	322 (354)	−46.7 (−125.2, 31.8)		
Year 4	277 (301)	−91.6 (−170.2, −13.1)		

a Analysis of covariance, modeling minutes of physical activity per week as outcome variable, subject as a fixed blocking term, and survey time as fixed exposure variable.

b Estimated increase (decrease) in minutes of physical activity per week from baseline to year 1 and year 4 follow-up surveys, respectively.

c Global test assessing if minutes of physical activity per week differed across any of the 3 survey times.

d Paired *t* tests assessing in turn whether minutes of physical activity per week differed at baseline survey compared with year 1 survey, at baseline survey compared with year 4 survey, and at year 1 survey compared with year 4 survey.

### Subgroup analyses

**Figure 3.**
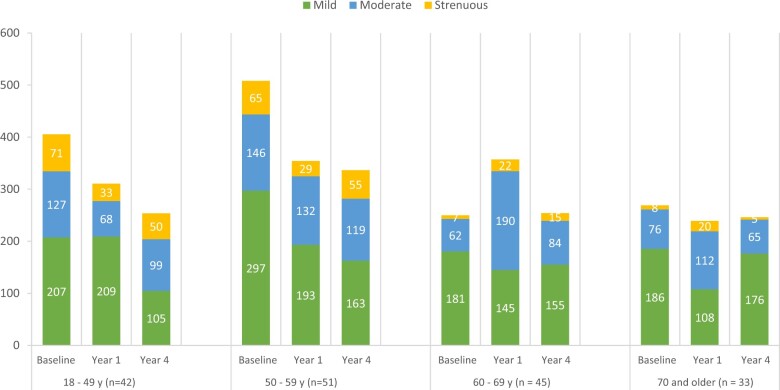
Average physical activity over time by age group. Average minutes per week of mild-intensity, moderate-intensity, and strenuous physical activity by age group at baseline, year 1, and year 4 timepoints.

**Figure 4.**
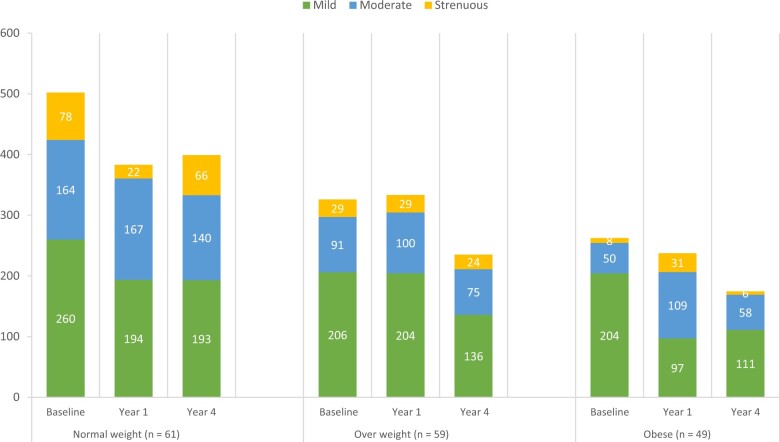
Average physical activity over time by body mass index (BMI) group. Average minutes per week of mild-intensity, moderate-intensity, and strenuous physical activity by BMI group at baseline, year 1, and year 4 timepoints.

Among participants aged 18-49 years, mild-intensity (ANCOVA *P* = .05) and moderate-intensity (ANCOVA *P* = .02) physical activity decreased over time, as did strenuous physical activity specifically from the baseline to year 1 timepoint (paired *t* test *P* = .04) (Supplementary Table 2, available online) (Figure 3). Among participants aged 50 years or older, neither mild-intensity, moderate-intensity, nor strenuous physical activity levels experienced a statistically significant decrease over time (Supplementary Table 2, available online) (Figure 3). Among participants with a normal BMI, strenuous physical activity levels decreased over time (ANCOVA *P* = .002), as did for those with obesity (ANCOVA *P* = .01). Among those with an overweight BMI, neither mild-intensity, moderate-intensity, nor strenuous physical activity levels experienced a statistically significant decrease over time. These findings are shown in Supplementary Table 3 (available online) and Figure 4.

## Discussion

In this cohort of breast cancer survivors, we report a trend-level decrease in total physical activity over time. We found that mild-intensity physical activity decreases most over time and that the statistically significant decrease in total physical activity from baseline to year 1 is primarily driven by a decrease in mild-intensity physical activity. As we did not include participants who had had recurrence of their breast cancer or metastatic disease, we hypothesize that by the year 4 survey, most participants should have recovered from the acute toxicities of surgery, radiation, and/or chemotherapy; nevertheless, physical activity at year 4 did not return to baseline levels in 45.6% of participants. We hypothesize that the mechanism of this decrease in mild physical activity may result from persistent side effects of maintenance treatments (such as arthralgias from aromatase inhibitors) and/or establishment of new lifestyle habits during acute cancer treatment that persist beyond the period of intense treatment. The decline in mild-intensity physical activity is notable because this represents the majority of physical activity reported at all timepoints. However, predictably, the percentage of participants meeting guidelines (a summation of average moderate-intensity and/or strenuous physical activity) remained fairly constant over time. It’s important to note that nearly one-third of participants did not meet guidelines at any timepoint.

Similar to the cohort as a whole, in age-related subgroup analyses, there was no statistically significant change in total physical activity over time within each age group. Younger participants (aged younger than 50 years) experienced important decrements in mild-intensity and moderate-intensity physical activity across all timepoints, as well as decrements in strenuous physical activity from baseline to year 1. This suggests that younger breast cancer survivors are particularly vulnerable to reductions in physical activity up to 4 years after diagnosis.

There was no statistically significant change in physical activity over time across BMI groups. Interestingly, strenuous physical activity decreased most notably in those with a normal BMI, who started at higher physical activity levels relative to those with an elevated BMI. Previous research has shown that breast cancer survivors who decrease their physical activity from prediagnosis to posttreatment had higher levels of fatigue and depression and worse quality of life, even more so than those with low physical activity levels throughout (25).

The National Comprehensive Cancer Network Survivorship Guideline highlights specific strategies to increase physical activity in breast cancer survivors including the following: 1) physician recommendation; 2) referral to trained personnel or exercise specialist if available; 3) supervised exercise program or classes; 4) telephone counseling; 5) motivational interviewing; 6) evaluation of readiness to change, importance of change, self-efficacy; 7) cancer survivor-specific materials and resources; 8) setting short- and long-term goals; 9) use of a pedometer or wearable fitness tracker to monitor activity and obtain at least 7000-10 000 steps per day; and 10) encouraging social support (exercise buddy, group) (16). Previous systematic reviews have suggested yoga, aerobic resistance exercise, and aerobic yoga as particularly beneficial modalities for mitigating cancer-related fatigue among women with breast cancer (26). National Institutes of Health funding of research related to physical activity has increased from $392 million in 2016 to $677 million in 2022 (27) though with little change in physical activity engagement; as such, further research targeting clinical implementation of physical activity guidelines and specific benefits of interventions among those at highest risk of inactivity is warranted. As we found that statistically significant decrements in physical activity amounts occurred from baseline to year 4, our findings support a prolonged duration for physical activity interventions.

The longitudinal nature of the study, which allowed for comparison of physical activity levels over time, is a distinct strength. Limitations of this study include a modest sample size, which reduced power to detect statistically significant changes in physical activity over time. This was especially true within age and BMI subgroups. The proportion of participants responding to all 3 surveys was relatively low, increasing the possibility of response bias. Additionally, we performed multiple subgroup analyses, increasing the likelihood of falsely rejecting the null hypothesis for some. The population was predominantly White and non-Hispanic, and all were seen in an academic medical center in the Midwest United States, which may limit the generalizability of the findings of the present study. As such, research across multiple centers will be important to evaluate if these results are reproducible across a more diverse population. In addition, given the long period of time between surveys, our data may be influenced by recall bias, as participants may not accurately recall the amount and/or intensity of physical activity participation in preceding months, and a social desirability bias, as most people know that physical activity is beneficial and may overreport their participation. Future studies with more objective measures of physical activity, such as wearable activity trackers, may mitigate some of these biases. Future directions will also include additional surveys of our current study population to assess later changes in physical activity levels, as well as interventions that may support physical activity in breast cancer survivors.

Overall, these data suggest that, despite frequent interactions with the health-care system (opportunities for counseling about the importance of physical activity during survivorship), breast cancer survivors reduce their overall physical activity over 4 years after diagnosis. National health survey data suggest that the percentage of people in the United States who meet aerobic physical activity guidelines stays fairly constant across age categories (28), so this decline may be specific to cancer survivors. The specific barriers to physical activity participation have not been thoroughly explored. Previous studies have suggested that lack of motivation and/or interest and symptoms of pain and fatigue are barriers to physical activity guideline adherence in cancer survivors (29,30). An additional study among breast cancer survivors found that the most frequently reported perceived barriers to physical activity were physical injury and symptoms (31). At least short term, wearable activity trackers coupled with health coaching has decreased inactivity in breast cancer survivors (32). A recent study also showed that a telephone-based weight loss intervention can be effective in supporting weight loss in breast cancer survivors (33), and similar interventions should be studied in other settings to increase physical activity. Focusing solely on meeting or not meeting moderate- and strenuous-intensity physical activity guidelines may not fully capture changes in activity over time, as we found that mild-intensity physical activity, which has not been the focus of national guidelines, was most likely to decrease.

## Supplementary Material

pkad056_Supplementary_Data

## Data Availability

Unidentifiable clinical and survey data are available when consistent with the informed consent document signed by participants in this study. Requests for data should be sent to ruddy.kathryn@mayo.edu for review by the executive committee of the Mayo Clinic Breast Disease Registry.
