# Supplementary material for: Changes in amount and intensity of physical activity over time in breast cancer survivors
Source: JNCI Cancer Spectr. 2023 Aug 10;7(5):pkad056. doi: 10.1093/jncics/pkad056 (PMC10471529; doi:10.1093/jncics/pkad056)
Supplement: pkad056_Supplementary_Data [file pkad056_supplementary_data.pdf]

Supplementary Table 1. Characteristics of survey respondents and compliance with physical activity guidelines across all three surveys.

	Meeting guidelines across all surveys			
	Never (N=51)	Always (N=18)	Total (N=69)	P-value
**Age at diagnosis**, n (%)				0.008[1]
18-39	4 (7.8%)	1 (5.6%)	5 (7.2%)	
40-49	5 (9.8%)	5 (27.8%)	10 (14.5%)	
50-59	8 (15.7%)	7 (38.9%)	15 (21.7%)	
60-69	22 (43.1%)	1 (5.6%)	23 (33.3%)	
>= 70	12 (23.5%)	4 (22.2%)	16 (23.2%)	
**Gender**, n (%)				1.000[1]
Female	49 (96.1%)	18 (100.0%)	67 (97.1%)	
Male	2 (3.9%)	0 (0.0%)	2 (2.9%)	
**Race**, n (%)				1.000[1]
African/African American/Black	2 (3.9%)	0 (0.0%)	2 (2.9%)	
Other	1 (2.0%)	0 (0.0%)	1 (1.4%)	
White	48 (94.1%)	18 (100.0%)	66 (95.7%)	
**Ethnicity**, n (%)				0.603[1]
Hispanic/Latino	1 (2.0%)	1 (5.6%)	2 (2.9%)	
Not Hispanic/Latino	49 (96.1%)	17 (94.4%)	66 (95.7%)	
Unknown/choose not	1 (2.0%)	0 (0.0%)	1 (1.4%)	
**BMI**, n (%)				0.001[1]
18.5-24.9	10 (19.6%)	10 (55.6%)	20 (29.0%)	
25.0-29.9	17 (33.3%)	7 (38.9%)	24 (34.8%)	
30.0+	24 (47.1%)	1 (5.6%)	25 (36.2%)	
**Highest level of education**, n (%)				0.924[1]
Bachelor's degree	7 (13.7%)	4 (22.2%)	11 (15.9%)	
Graduate school	19 (37.3%)	7 (38.9%)	26 (37.7%)	
High school graduate, GED	7 (13.7%)	2 (11.1%)	9 (13.0%)	
Some college or Associate’s degree, but not Bachelor’s degree	15 (29.4%)	4 (22.2%)	19 (27.5%)	
Vocational education beyond high school	3 (5.9%)	1 (5.6%)	4 (5.8%)	
**Hypercholesterolemia**, n (%)				0.779[1]
No	30 (60.0%)	12 (66.7%)	42 (61.8%)	
Yes	20 (40.0%)	6 (33.3%)	26 (38.2%)	
Missing	1	0	1	

	Meeting guidelines across all surveys			
	Never (N=51)	Always (N=18)	Total (N=69)	P-value
**Heart disease**, n (%)				1.000[1]
No	47 (95.9%)	16 (94.1%)	63 (95.5%)	
Yes	2 (4.1%)	1 (5.9%)	3 (4.5%)	
Missing	2	1	3	
**Hypertension**, n (%)				0.153[1]
No	27 (54.0%)	13 (76.5%)	40 (59.7%)	
Yes	23 (46.0%)	4 (23.5%)	27 (40.3%)	
Missing	1	1	2	
**Stroke**, n (%)				
No	49 (100.0%)	17 (100.0%)	66 (100.0%)	
Missing	2	1	3	
**Diabetes**, n (%)				0.098[1]
No	40 (81.6%)	17 (100.0%)	57 (86.4%)	
Yes	9 (18.4%)	0 (0.0%)	9 (13.6%)	
Missing	2	1	3	
**Lung disease**, n (%)				0.270[1]
No	39 (81.3%)	16 (94.1%)	55 (84.6%)	
Yes	9 (18.8%)	1 (5.9%)	10 (15.4%)	
Missing	3	1	4	
**Liver disease**, n (%)				0.452[1]
No	48 (98.0%)	16 (94.1%)	64 (97.0%)	
Yes	1 (2.0%)	1 (5.9%)	2 (3.0%)	
Missing	2	1	3	
**Kidney disease**, n (%)				0.561[1]
No	45 (93.8%)	17 (100.0%)	62 (95.4%)	
Yes	3 (6.3%)	0 (0.0%)	3 (4.6%)	
Missing	3	1	4	
**Surgery type**, n (%)				0.056[1]
BL Mastectomy	10 (19.6%)	9 (50.0%)	19 (27.5%)	
Biopsy only/other/unknown	4 (7.8%)	1 (5.6%)	5 (7.2%)	
Lumpectomy	27 (52.9%)	4 (22.2%)	31 (44.9%)	
UL Mastectomy	10 (19.6%)	4 (22.2%)	14 (20.3%)	
**Radiotherapy**, n (%)				0.159[1]
No	17 (33.3%)	10 (55.6%)	27 (39.1%)	

	Meeting guidelines across all surveys			
	Never (N=51)	Always (N=18)	Total (N=69)	P-value
Yes	34 (66.7%)	8 (44.4%)	42 (60.9%)	
**Chemotherapy**, n (%)				0.743[1]
No	39 (76.5%)	15 (83.3%)	54 (78.3%)	
Yes	12 (23.5%)	3 (16.7%)	15 (21.7%)	
**Hormone therapy**, n (%)				0.565[1]
No	21 (41.2%)	10 (55.6%)	31 (44.9%)	
Unknown	1 (2.0%)	0 (0.0%)	1 (1.4%)	
Yes	29 (56.9%)	8 (44.4%)	37 (53.6%)	

[1]Fisher Exact p-value;

Supplementary Table 2. Associations of changes in mild, moderate, strenuous and total minutes of physical activity per week across survey times, by categories of age.

	Age 18-49 (N=42)			Age 50-59 (N=51)			Age 60-69 (N=45)			Age 70+ (N=33)		
Minutes of physical activity per week	Mean (SD)	Global p-value[1]	Pairwise p-values[2]	Mean (SD)	Global p-value[1]	Pairwise p-values[2]	Mean (SD)	Global p-value[1]	Pairwise p-values[2]	Mean (SD)	Global p-value[1]	Pairwise p-values[2]
Mild		0.05	0.97, 0.05, 0.005		0.26	0.34, 0.15, 0.50		0.58	0.36, 0.48, 0.72		0.18	0.06, 0.84, 0.16
Baseline	207 (335)			297 (736)			181 (219)			186 (220)		
Year 1	209 (237)			193 (257)			145 (145)			108 (129)		
Year 4	105 (112)			163 (271)			155 (152)			176 (261)		
Moderate		0.02	0.01, 0.22, 0.11		0.73	0.72, 0.43, 0.63		0.13	0.12, 0.14, 0.21		0.11	0.15, 0.65, 0.03
Baseline	127 (121)			146 (279)			62 (92)			76 (128)		
Year 1	68 (89)			132 (140)			190 (539)			112 (97)		
Year 4	99 (107)			119 (144)			84 (136)			65 (91)		
Strenuous		0.06	0.04, 0.13, 0.28		0.16	0.06, 0.57, 0.22		0.36	0.20, 0.14, 0.56		0.10	0.11, 0.55, 0.10
Baseline	71 (105)			65 (119)			7 (21)			8 (24)		
Year 1	33 (77)_			29 (73)			22 (74)			20 (47)		
Year 4	50 (69)			55 (134)			15 (41)			5 (19)		
Total		0.07	0.09, 0.02, 0.16		0.07	0.26, 0.10, 0.78		0.07	0.22, 0.92, 0.23		0.07	0.54, 0.68, 0.89
Baseline	405 (404)			508 (924)			250 (246)			269 (238)		
Year 1	311 (253)			354 (310)			357 (537)			239 (164)		
Year 4	254 (173)			337 (423)			254 (238)			246 (276)		

1. Global test assessing if minutes of physical activity per week differed across any of the three survey times. Analysis of covariance, modeling minutes of physical activity per week as outcome variable, subject as a fixed blocking term and survey time as fixed exposure variable.

2. Paired t-tests assessing in turn whether minutes of physical activity per week differed at baseline survey compared to Year 1 survey, at baseline survey compared to Year 4 survey, and at Year 1 survey compared to Year 4 survey.

Supplementary Table 3. Associations of changes in mild, moderate, strenuous and total minutes of physical activity per week across survey times, by categories of BMI.

	Normal (BMI 18.5-24.9, N=61)			Overweight (BMI 25.0-29.9, N=59)			Obese (BMI 30.0+, N=49)		
Minutes of physical activity per week	Mean (SD)	Global p-value[1]	Pairwise p-values[2]	Mean (SD)	Global p-value[1]	Pairwise p-values[2]	Mean (SD)	Global p-value[1]	Pairwise p-values[2]
Mild		0.29	0.25, 0.17, 0.99		0.22	0.97, 0.11, 0.08		0.27	0.23, 0.27, 0.55
Baseline	260 (458)			206 (304)			204 (605)		
Year 1	194 (210)			204 (256)			97 (96)		
Year 4	193 (284)			136 (162)			111 (139)		
Moderate		0.86	0.96, 0.43, 0.66		0.42	0.66, 0.35, 0.19		0.07	0.09, 0.64, 0.10
Baseline	164 (259)			91 (116)			50 (94)		
Year 1	167 (434)			100 (114)			109 (227)		
Year 4	140 (157)			75 (97)			58 (90)		
Strenuous		0.002	<0.001, 0.45, 0.007		0.88	0.96, 0.53, 0.70		0.01	0.04, 0.54, 0.03
Baseline	78 (129)			29 (53)			8 (26)		
Year 1	22 (53)			29 (79)			31 (77)		
Year 4	66 (127)			24 (54)			6 (20)		
Total		0.07	0.22, 0.10, 0.82		0.07	0.90, 0.09, 0.03		0.07	0.80, 0.31, 0.12
Baseline	502 (696)			325 (356)			262 (605)		
Year 1	383 (454)			333 (291)			237 (267)		
Year 4	399 (398)			236 (228)			175 (165)		

1. Global test assessing if minutes of physical activity per week differed across any of the three survey times. Analysis of covariance, modeling minutes of physical activity per week as outcome variable, subject as a fixed blocking term and survey time as fixed exposure variable.
2. Paired t-tests assessing in turn whether minutes of physical activity per week differed at baseline survey compared to Year 1 survey, at baseline survey compared to Year 4 survey, and at Year 1 survey compared to Year 4 survey.
